# Exploring the Anticancer Potential of *Origanum majorana* Essential Oil Monoterpenes Alone and in Combination against Non-Small Cell Lung Cancer

**DOI:** 10.3390/nu15235010

**Published:** 2023-12-04

**Authors:** Kholoud Arafat, Aya Mudhafar Al-Azawi, Shahrazad Sulaiman, Samir Attoub

**Affiliations:** 1 Department of Pharmacology & Therapeutics, College of Medicine & Health Sciences, United Arab Emirates University, Al-Ain 15551, United Arab Emirates; kholoud.arafat@uaeu.ac.ae (K.A.); 201870001@uaeu.ac.ae (A.M.A.-A.); sharazadjeffy@uaeu.ac.ae (S.S.); 2 ASPIRE Precision Medicine Research Institute Abu Dhabi, United Arab Emirates University, Al-Ain 15551, United Arab Emirates

**Keywords:** NSCLC, monoterpenes, terpinen-4-ol, sabinene hydrate, α-terpinene, *γ*-terpinene, survivin, tumor growth

## Abstract

Lung cancer is the second most commonly diagnosed cancer and has the highest mortality rate worldwide despite the remarkable advances in its treatment. *Origanum majorana* Essential Oil (OMEO) has been shown to be effective against non-small cell lung cancer (NSCLC) cells, decreasing their viability and colony growth in vitro, as well as inhibiting tumor growth in chick embryo chorioallantoic membranes (CAM) and nude mice in vivo. OMEO is mainly composed of four monoterpenes, namely terpinen-4-ol, sabinene hydrate, α-terpinene, and *γ*-terpinene. In this study, we aimed to investigate the potential anticancer effects of these monoterpenes, either alone or in combination, on NSCLC. Our findings indicate that these four monoterpenes significantly decreased NSCLC cell viability in a concentration-dependent manner, reduced their colony growth in vitro, and also downregulated survivin expression in these cells. Moreover, different combined mixtures of these monoterpenes further enhanced their anticancer effects on cellular viability, with a terpinen-4-ol and sabinene hydrate combination being the most potent. We also found that terpinen-4-ol, in combination with sabinene hydrate, markedly enhanced the anticancer effect of the individual monoterpenes on NSCLC viability within a shorter treatment duration through, at least in part, survivin downregulation. Furthermore, this combination enhanced the inhibition of colony growth in vitro and the tumor growth of NSCLC cells xenografted onto chick embryo CAM in vivo. Altogether, our study highlights the potential of these monoterpenes for use in further pre-clinical investigations against various cancer hallmarks.

## 1. Introduction

In the last decades, significant advances have been attained in understanding lung cancer progression, in addition to the incorporation of various targeted and immunotherapies in the management protocols [1]. However, according to the global official reports from the International Agency for Research on Cancer in 2020, lung cancer is the second most commonly diagnosed type of cancer, with approximately 2.2 million new cases, and also the most common cause of cancer mortality with approximately 1.8 million deaths [2]. Most of the new lung cancer cases are diagnosed at advanced stages, which further complicates treatment outcomes and causes a steep drop in the 5-year relative survival rate to only 7% [3,4]. Moreover, the side effects of the current treatment protocols, the development of resistance, the ineligibility of new treatments, and the high cost can be reasons for the continuous burden of lung cancer [1,5,6]. Thus, efforts are ongoing to develop new drugs and treatment strategies targeting critical molecular pathways in lung cancer to improve patient outcomes.

**Figure 1:**
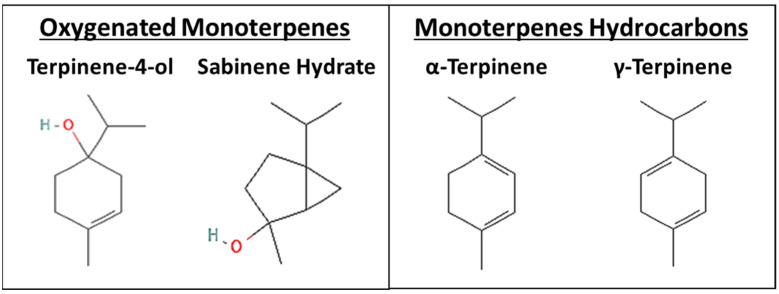
Chemical structure of the most abundant oxygenated monoterpenes and monoterpene hydrocarbons in OMEO (National Centre for Biotechnology Information. PubChem Compound Summary).

Essential oils (EOs) are complex aromatic plant metabolites that have gained interest in cancer treatment worldwide. The complex composition of these EOs explains their multiple mechanisms of action and makes them effective multi-target agents that may optimize the anticancer response [7]. *Origanum majorana* is a widespread worldwide plant whose essential oil has been investigated by our group on NSCLC. We previously reported that OMEO significantly decreased the viability and clonogenic growth of the NSCLC cells. Moreover, OMEO significantly slowed down the growth of NSCLC tumor xenografts in the CAM and nude mice models by downregulating survivin expression. OMEO was also shown to reduce migration and invasion of NSCLC cells in vitro and metastasis in vivo, making it and its constituents promising candidates for further investigation in cancer treatment [8]. The main chemical components of OMEO are the oxygenated monoterpenes, including terpinen-4-ol and sabinene hydrate, and monoterpene hydrocarbons, including α-terpinene and *γ*-terpinene (Figure 1) [9,10,11]. These monoterpenes have been shown to have promising anticancer effects on various cancer cells. Specifically, terpinen-4-ol was reported to induce apoptosis in colorectal, pancreatic, prostate, gastric, and NSCLC cells [12,13]. α-terpinene inhibits the proliferation of MCF-7 human breast cancer cells and promotes cancer cell death by inducing oxidative damage in cancer cells in vitro [14]. Similarly, *γ*-terpinene was reported to have anticancer effects on B16-F10 mouse melanoma cells but not on HepG2 human hepatocellular carcinoma or K562 human chronic myelocytic leukemia [15].

To determine whether the four most abundant terpenes found in OMEO mediate its previously reported anticancer activity, we investigated the potential of these monoterpenes to inhibit NSCLC cell growth using the A549 and LNM35 cell lines. Cellular viability, clonogenic growth, and survivin expression in vitro, as well as tumor growth in chick embryo CAM in vivo, were evaluated upon treatment with these monoterpenes alone and in combination.

## 2. Materials and Methods

### 2.1. Cell Culture and Reagents

Human NSCLC A549 cells were purchased from the American Type Culture Collection (ATCC, Manassas, VA, USA), and LNM35 cells were generously provided by Prof. Takahashi. Both cell lines were cultured in RPMI-1640 medium supplemented with 10% FBS (Hyclone, Cramlington, UK) and 1% of antibiotic/antimycotic solution 100X (Hyclone, Cramlington, UK). The cells were incubated in humidified incubator set at 5% CO_2_ and 37 °C. The cells were sub-cultured once a week upon reaching 95% confluency, and the cell culture medium was changed every 3 days.

OMEO was purchased from Pranarom Scientific Aromatherapy (Paris, France). Based on the composition provided by the company, four monoterpenes are the main constituents of this essential oil. They are found at a concentration of 21.8% for terpinene-4-ol, 9.7% for sabinene, 10.51% for alpha-terpinene, and 16.37% for gamma-terpene. Terpinen-4-ol, *ϒ*-terpinene, and sabinene hydrate were sourced from HWI Pharma Services (Ruelzheim, Germany), and α-terpinene was purchased from Sigma Aldrich (Saint Louis, MO, USA).

### 2.2. Cellular Viability

NSCLC cells were seeded in 96-well plates at a density of 5000 cells/well. After 24 h, the seeded cells were treated in duplicate with increasing concentrations of monoterpenes for 24, 48, and 72 h. After the treatment period, cellular viability was determined using the CellTiter-Glo Luminescent Cell Viability Assay, which measures the amount of ATP in metabolically active cells. The luminescent signal was quantified using the GLOMAX Luminometer (Promega Corporation, Madison, WI, USA). The viability of treated cells was presented as a percentage (%) by comparing it to the viability of control cells, which was assumed to be 100%.

### 2.3. Colony Growth Assay

A549 and LNM35 cells were seeded in 6-well plates at a density of 50 and 100 cells per well, respectively. The cells were allowed to grow into colonies for 10–14 days in a humidified incubator set at 5% CO_2_ and 37 °C. Subsequently, the formed colonies were treated in duplicate with two different concentrations of each monoterpene at three-day intervals over a period of seven days. Control colonies were treated with the same volume of the vehicle used to dissolve the terpenes. Afterward, colonies were fixed and stained for 2 h with 0.5% crystal violet dissolved in 50% methanol (*v*/*v*). Colonies were washed with tap water and allowed to dry. Colonies containing more than 50 cells were counted using an inverted microscope. The colony percentage (%) was determined by comparing the number of colonies in treated groups to the number of colonies in the control, which was assumed to be 100%.

### 2.4. In Ovo Tumor Growth Assay

As previously described [8], fertilized leghorn eggs were incubated at 37.5 °C and 50% humidity in the egg incubator for the first three days of embryonic development. On embryonic day 3 (E3), 1.5–2 mL of albumin was aspirated through a hole opposite the round, wide egg side. A 1 cm^2^ window was created in the eggshell above the upper CAM and sealed with Suprafilm. At E9, cells were trypsinized, counted, and suspended in 80% Matrigel at a density of 1 × 10^6^ cells/100 µL for A549 and 0.1 × 10^6^ cells/100 uL for LNM35. A 100 µL inoculum was added to the CAM of each egg, for a total of 9–12 eggs per condition. At E11, the treatment began by dropping 100 µL of the treatment solution into the tumors. The treatment was repeated three times. At E17, the upper portion of the CAM was removed, washed with PBS, and weighed. The eggs were randomly assigned to the treatment groups, but the experimenter was not blinded to the groups.

### 2.5. Western Blot

NSCLC cells were seeded in 6-well plates. In this first part of the experiments, cells were treated with each monoterpene for 24 and 48 h. In the second part of the experiments, the cells were treated with a combination of monoterpenes for 30 min and 2 and 6 h. At the designated time, cells were harvested and subjected to lysis using RIPA buffer, containing 25 mM Tris. HCl at pH 7.6, 1% Nonidet P-40, 1% sodium deoxycholate, 0.1% SDS, 0.5% protease inhibitor blend, 1% PMSF, and 1% phosphatase inhibitor blend. Then, centrifugation at 14,000 rpm for 20 min at 4 °C facilitated the recovery of whole cell extracts by discarding non-soluble debris. BCA Protein Assay kit from Thermo Fisher Scientific (Waltham, MA, USA) was employed to determine protein concentrations.

Proteins (20 µg) were separated by SDS-PAGE gel to determine the expression of survivin. The proteins were then wet-transferred onto a nitrocellulose membrane for 90 min. The membranes were blocked with 5% non-fat milk in TBST (*w*/*v*), followed by washing and incubation with primary antibody at 4 °C overnight. Blots were then washed and incubated with appropriate secondary antibodies. Visualization of the immunoreactive bands was achieved using the ECL substrate from Thermo Fisher Scientific and captured using the LiCOR C-DiGit Blot Imaging System, Version 3. For quantification, densitometric analyses were conducted using the scanner’s inherent software. All band intensity values were then normalized to the intensities of their corresponding β-actin signals.

### 2.6. Statistical Analysis

All the experiments were repeated at least three times except for the in ovo tumor growth assay. Results are presented as means ± S.E.M of the raw data. GraphPad Prism for Windows, version 9 (GraphPad Software, San Diego, CA, USA) was used to perform the statistical analysis. One-way ANOVA followed by Dunnett’s multiple comparison was applied to the data for cellular viability and colony growth. One-way ANOVA followed by Tukey’s multiple comparison was used for CAM data and combination. Statistical significance was denoted as follows: * *p* < 0.05, ** *p* < 0.01, *** *p* < 0.001, and **** *p* < 0.0001.

## 3. Results and Discussions

### 3.1. The Four Monoterpenes Inhibited NSCLC Cellular Viability and Colony Growth In Vitro

**Figure 2:**
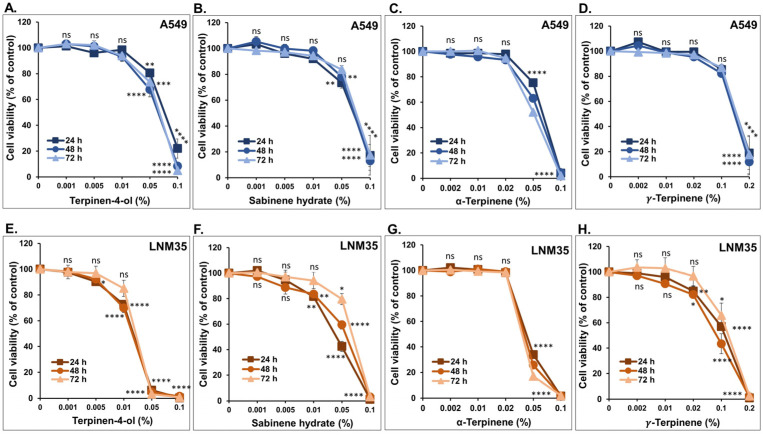
Effect of the four monoterpenes on NSCLC cellular viability in vitro. A549 (**A**–**D**) and LNM35 (**E**–**H**) cells were treated with increasing concentrations of terpinen-4-ol, sabinene hydrate, α-terpinene, or *γ*-terpinene for 24, 48, and 72 h. Cellular viability was determined as described in the materials and methods. Experiments were repeated at least three times. Shapes represent mean and bars represent S.E.M. * *p* < 0.05, ** *p* < 0.01, *** *p* < 0.001, and **** *p* < 0.0001; ns—non-significant.

The anticancer effect of the most abundant monoterpenes that constitute the OMEO was first assessed on the cellular viability of NSCLC cells, namely A549 and LNM35, after 24–72 h of treatment with concentrations ranging from 0.001% to 0.2%. As shown in Figure 2, the four monoterpenes, terpinen-4-ol, sabinene hydrate, α-terpinene, or *γ*-terpinene, reduced the cellular viability of A549 (Figure 2A–D) and LNM35 (Figure 2E–H) in a concentration-dependent manner. No major differences were observed in the viability between 24, 48, and 72 h of treatment. At 48 h, the IC50 concentrations affecting cell viability in A549 and LNM35 were observed as follows: terpinen-4-ol at 0.06% for A549 and 0.02% for LNM35, sabinene hydrate at 0.06% for A549 and 0.05% for LNM35, α-terpinene at 0.06% for A549 and 0.04% for LNM35, and γ-terpinene at 0.13% for A549 and 0.08% for LNM35.

**Figure 3:**
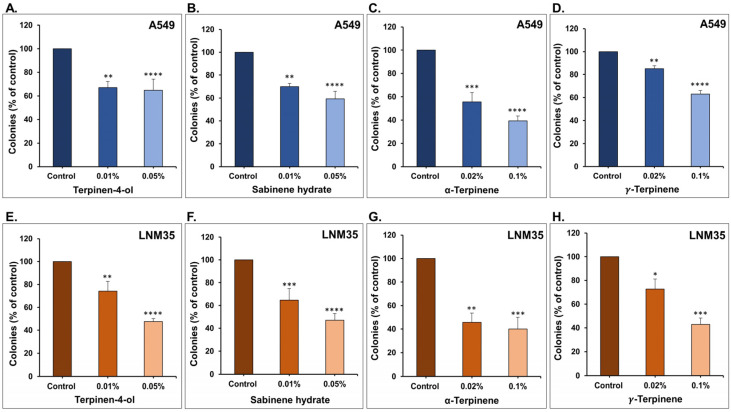
Effect of the four monoterpenes on NSCLC colony growth. A549 and LNM35 formed colonies were treated every three days for 1 week with terpinen-4-ol (**A**,**E**), sabinene hydrate (**B**,**F**), α-terpinene (**C**,**G**), or *γ*-terpinene (**D**,**H**) for 1 week. Colonies were fixed, stained, and counted as described in the materials and methods. Experiments were repeated at least three times. Columns represent mean; bars represent S.E.M. * *p* < 0.05, ** *p* < 0.01, *** *p* < 0.001, and **** *p* < 0.0001.

Subsequently, we aimed to further investigate the in vitro activity of these monoterpenes using a colony growth assay. For this, the formed colonies of A549 and LNM35 were treated with two different concentrations of terpinen-4-ol, sabinene hydrate, α-terpinene, or *γ*-terpinene for 1 week. These four monoterpenes markedly decreased the total number of A549 (Figure 3A–D) and LNM35 (Figure 3E–H) colonies.

Several reports have been published documenting the impact of terpinen-4-ol on different types of cancer. However, to the best of our knowledge, there is limited research on α-terpinene, *γ*-terpinene, or sabinene hydrate. Our results with terpinen-4-ol align with those of Wu et al. (2012), who reported a significant concentration-dependent reduction in the viability of NSCLC A549 and CL1-0 cells upon treatment with terpinen-4-ol (0.02–0.1%) for 24 h. The IC_50_ was reported to be 0.052 and 0.046% in the aforementioned cell lines, respectively [13]. Terpinen-4-ol, at concentrations between 0.005–0.1%, was also found to significantly decrease the viability of colorectal, pancreatic, prostate, and gastric cancer cell lines in a concentration-dependent manner after 72 h of incubation [12]. Moreover, a concentration-dependent decrease in viability was reported in colorectal cancer cells HCT116 and RKO, when treated with terpinen-4-ol at concentrations ranging from 100 to 10,000 µM [16]. This compound also reduced the proliferation of pancreatic cancer cells AsPC-1 and PANC-1 upon treatment with concentrations between 0.5 and 2 µM for either 24 or 48 h [17]. The in vitro anticancer effect of this monoterpene has been documented for melanoma cell lines M14, A375, and M14 ADR cells [18,19]. Additionally, it was reported that α-terpinene (0.13–6.75 µg/mL) inhibited the proliferation of breast cancer cells MCF-7, with a nearly 50% inhibition achieved at the concentration of 6.75 µg/mL [14]. *γ*-terpinene (10–100 µg/mL) caused around 10% and 20% inhibition in the cellular viability of the murine leukemic cell line WEHI-3 and the murine macrophage cell line RAW 264.7, respectively [20]. However, a concentration of 9.28 µg/mL of γ-terpinene reduced the viability of melanoma B16-F10 cells by 50% after a 72 h treatment [15]. On the other hand, γ-terpinene at concentrations of 1–10 mM did not have a significant impact on the viability of breast cancer cells MCF-7 [21]. In terms of colony growth, Cao et al. (2022) showed that terpinen-4-ol decreased the colony formation ability of pancreatic cells AsPC-1 and PANC-1 [17]. Furthermore, Cao et al. (2023) corroborated the inhibitory effect of terpinen-4-ol on colony formation in glioma cancer cells U251, T98, and LN229 [22].

### 3.2. The Four Monoterpenes Inhibited Survivin Expression In Vitro

**Figure 4:**
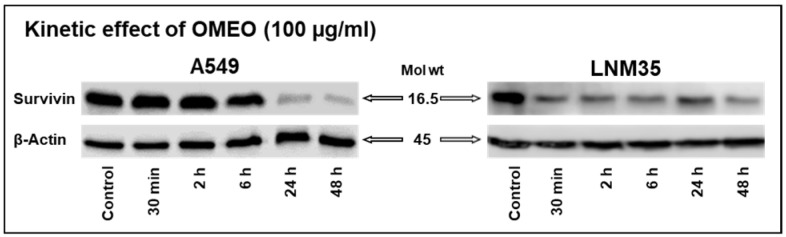
Kinetic effect of OMEO on survivin expression in NSCLC cells in vitro. Western blot analysis shows the level of survivin in A549 and LNM35 cells treated with OMEO 100 µg/mL for 0.5, 2, 6, 24, and 48 h. Experiments were repeated for at least three times.

We have previously reported that OMEO decreases the expression of survivin in tumors xenografted on chick embryo CAM and nude mice [8]. In this study, we sought to corroborate these findings by assessing in vitro the expression of survivin in NSCLC cells treated with OMEO for 0.5–48 h. As depicted in Figure 4, treatment with OMEO at a concentration of 100 µg/mL reduced the survivin expression in A549 and LNM35 cells.

**Figure 5:**
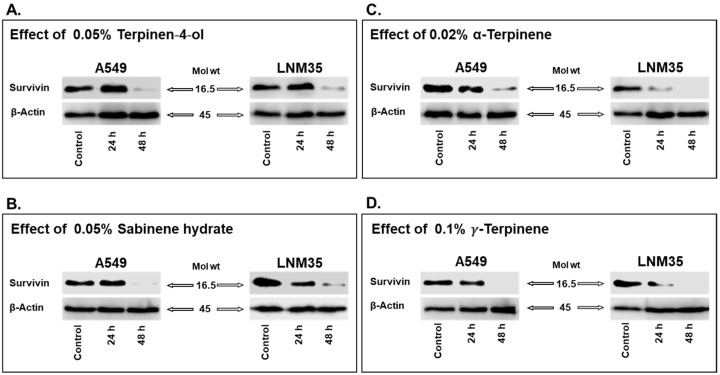
Effect of the four monoterpenes on survivin expression in NSCLC cells in vitro. A549 and LNM35 cells were treated with (**A**) terpinen-4-ol 0.05%, (**B**) sabinene hydrate 0.05%, (**C**) α-terpinene 0.02%, or (**D**) *γ*-terpinene 0.1% for 24 and 48 h. Experiments were repeated at least three times.

To assess whether the anticancer effect of the most abundant monoterpenes in OMEO is also mediated via survivin regulation in NSCLC cells, we treated our cells with the indicated concentrations of these monoterpenes for 24–48 h. As shown in Figure 5A–D, treatment with terpinene-4-ol, sabinene hydrate, α-terpinene, and γ-terpinene resulted in a reduction of survivin expression in both A549 and LNM35 cells. Notably, terpinene-4-ol reduced survivin expression starting at 48 h for A549 and LNM35 (Figure 5A), while sabinene hydrate decreased expression at 48 h for A549 and 24 h for LNM35 (Figure 5B). Almost similar results were obtained with α-terpinene (Figure 5C) and γ-terpinene (Figure 5D). Altogether, our results point to survivin as a potential target of these monoterpenes in NSCLC cells.

Survivin is the smallest protein among the inhibitor of apoptosis proteins (IAPs) family members [23]. Apart from inhibiting apoptosis and regulating cell division, survivin also contributes to autophagy, angiogenesis, and cellular stemness [24,25,26,27]. Survivin overexpression is common in various cancer types, including NSCLC, and has been linked to chemotherapy resistance, poor prognosis, metastasis, and a high risk of relapse [24,28]. Therefore, survivin is considered a promising target for cancer treatment. This study showed the downregulation of survivin by the two oxygenated monoterpenes and the two hydrocarbons’ monoterpenes in NSCLC cells. The inhibitory effect of terpinen-4-ol on survivin expression was documented once by Wu et al. (2012), who showed downregulation of survivin in NSCLC A549 and CL1-0 cells upon treatment with terpinen-4-ol (0.06–0.1%) for 24 h [13]. These data suggest that the anticancer effect of these monoterpenes is attributed, at least partly, to the downregulation of survivin expression.

### 3.3. The Anticancer Effect of Monoterpene Combinations on NSCLC In Vitro

**Figure 6:**
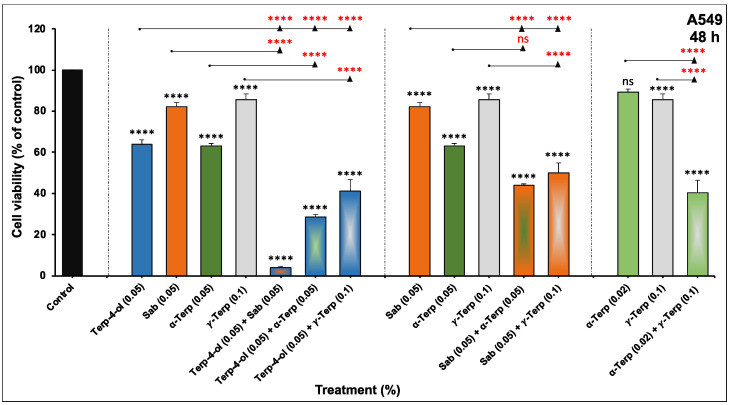
Effect of monoterpene combinations on NSCLC cellular viability in vitro. A549 cells were treated with different concentrations of monoterpenes alone or in combination for 48 h. Cellular viability was assessed using CellTiter-Glo Luminescent Assay as described in the materials and methods. Experiment was repeated at least three times. Columns represent means, and bars represent S.E.M. **** *p* < 0.0001. ns—non-significant. Black stars indicate significance in comparison to the control; red stars indicate significance between groups.

The potency of the EOs, including OMEO, may be due to the additive or synergistic interaction between the different monoterpenes and other constituents. To assess the anticancer potential of the four monoterpenes in combination, different mixture ratios were tested for their impact on the viability of A549 cells. As shown in Figure 6, all the combinations, “Terpinen-4-ol + Sabinene hydrate, Terpinen-4-ol + α-Terpinene, Terpinen-4-ol + γ-Terpinene, Sabinene hydrate + α-Terpinene, Sabinene hydrate + γ-Terpinene, and α-Terpinene + γ Terpinene”, significantly enhanced the effects of the single monoterpenes. Among these effective combinations, terpinen-4-ol + sabinene hydrate showed the highest inhibitory effect by inducing approximately a 90% reduction in cellular viability. Therefore, this combination was further investigated for its anticancer effect in NSCLC cells.

#### 3.3.1. Impact of Terpinen-4-ol in Combination with Sabinene Hydrate on NSCLC Cellular Viability and Colony Growth In Vitro

**Figure 7:**
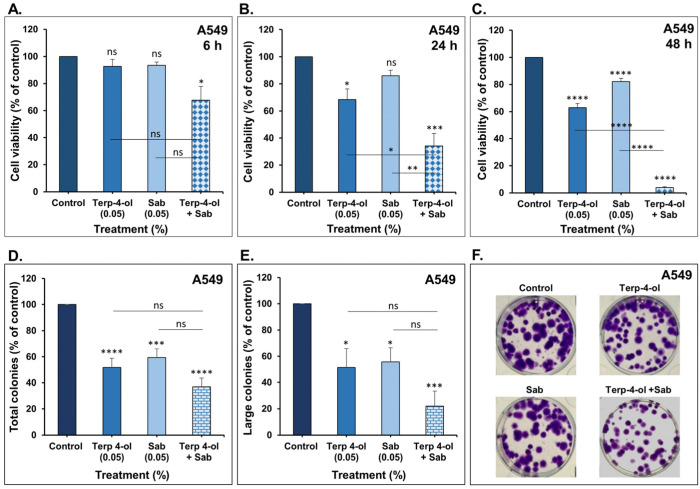
Effect of terpinen-4-ol in combination with sabinene hydrate on A549 viability and colony growth in vitro. (**A**–**C**) A549 cells were treated with terpinen-4-ol, sabinene hydrate, or a combination for 6, 24, and 48 h. Cellular viability was determined as described in the materials and methods. (**D**–**F**) A549 number of total and large colonies treated with terpinen-4-ol, sabinene hydrate, or a combination for 7 days. Experiments were repeated at least three times. Columns represent mean and bars are S.E.M. * *p* < 0.05, ** *p* < 0.01, *** *p* < 0.001, and **** *p* < 0.0001. ns—non-significant.

The effect of the combination of terpinen-4-ol and sabinene hydrate on cellular viability was assessed at short intervals in A549 and LNM35 cells. For A549 cells, neither of the monoterpenes alone significantly reduced cell viability after 6 h of treatment, but their combination led to a significant decrease of around 30% (Figure 7A). This inhibitory effect became more pronounced at 24 h and 48 h, with reductions of 60% and 96%, respectively (Figure 7B,C), indicating a time-dependent action of these combined monoterpenes. Additionally, the combination enhanced the inhibitory effect of each of the monoterpenes on both the total number and the size of A549 colonies, although statistical significance was not reached (Figure 7D–F).

**Figure 8:**
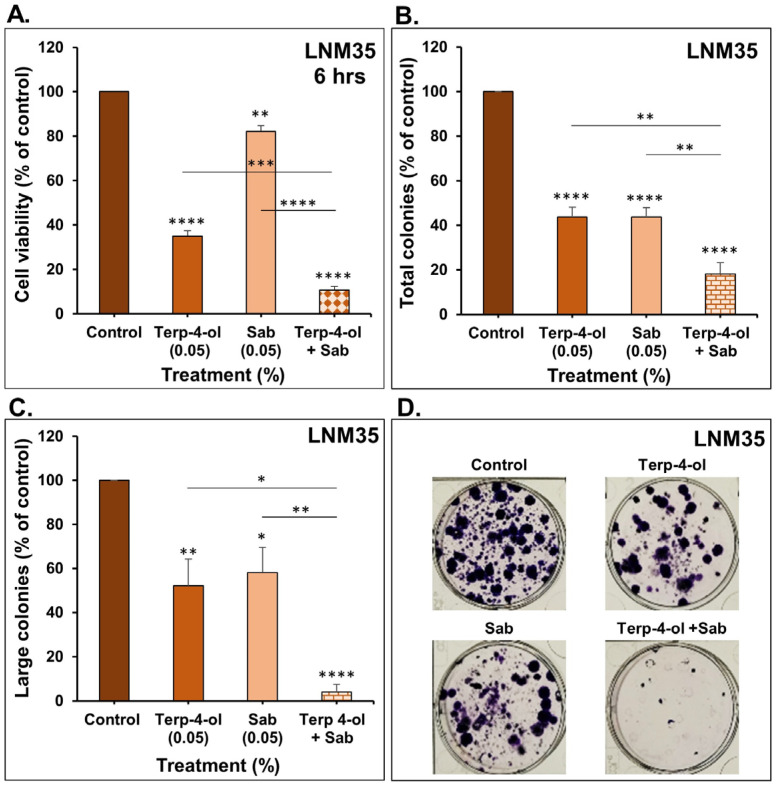
Effect of terpinen-4-ol in combination with sabinene hydrate on LNM35 viability and colony growth in vitro. (**A**) LNM35 cells were treated with terpinen-4-ol, sabinene hydrate, or a combination for 6 h. Cellular viability was determined as described in the materials and methods. (**B**–**D**) LNM35 number of total and large colonies treated with terpinen-4-ol, sabinene hydrate, or a combination for 7 days. Experiments were repeated at least three times. Columns represent mean and bars are S.E.M. * *p* < 0.05, ** *p* < 0.01, *** *p* < 0.001, and **** *p* < 0.0001.

Similarly, treatment of LNM35 cells with the combination of terpinen-4-ol and sabinene hydrate for 6 h significantly inhibited the viability compared to individual treatments (Figure 8A). Furthermore, this combination markedly enhanced the inhibitory effect of the individual treatments on the total number of LNM35 colonies, in addition to the large-size colonies (Figure 8B–D). Collectively, these findings suggest the potential of these monoterpenes as effective anticancer agents. To the best of our knowledge, this is the first study demonstrating the promising combinatory effect of these monoterpenes on cancer, including NSCLC cells. It has been reported that terpinen-4-ol exhibits synergistic growth inhibition on colorectal cancer cells when combined with Oxaliplatin and 5-FU and on pancreatic cancer cells when combined with Gemcitabine. Similar results were shown when combined with biological agents, such as anti-CD24 antibodies and anti-EGFR antibodies on colorectal and prostate cancer cells [12]. Therefore, our research sought to evaluate their mechanism of action and potential effects in vivo.

#### 3.3.2. Terpinen-4-ol in Combination with Sabinene Hydrate Decreased Survivin Expression

**Figure 9:**
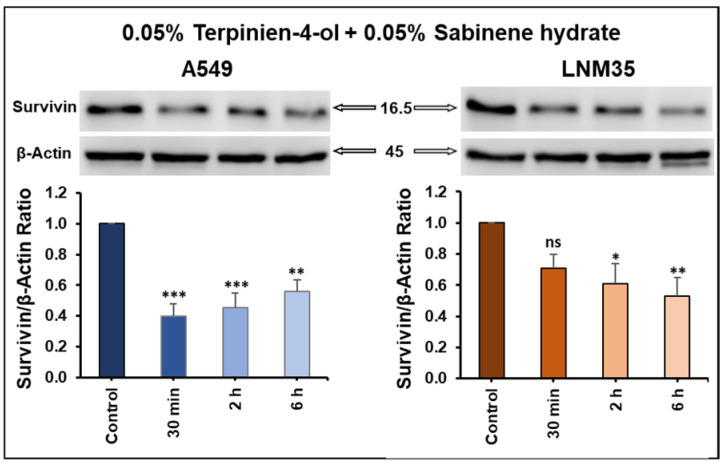
Effect of the combination of terpinen-4-ol and sabinene hydrate on survivin expression. Quantification of the Western blot showing the impact of terpinen-4-ol + sabinene hydrate on the expression of survivin in A549 and LNM35 cells. Columns represent means of at least three independent experiments; bars represent S.E.M. * *p* < 0.05. ** *p* < 0.01. *** *p* < 0.001. ns—non-significant.

We assessed the level of survivin in A549 and LNM35 cells treated with the combination of terpinen-4-ol and sabinene hydrate for short incubation times. As shown earlier in Figure 5, terpinen-4-ol or sabinene hydrate decreased survivin expression at 48 h, suggesting the absence of effect within shorter incubation periods. Figure 9 demonstrates that the combination of these two monoterpenes can downregulate survivin expression in both cell lines starting at 30 min after treatment. These results suggest that the anticancer effect of this combination is, at least partly, mediated through survivin downregulation. Given the crucial role of survivin in cancer cell survival and drug resistance, its downregulation is a promising target for anticancer therapy.

#### 3.3.3. Impact of Terpinen-4-ol in Combination with Sabinene Hydrate on NSCLC Tumors In Vivo

**Figure 10:**
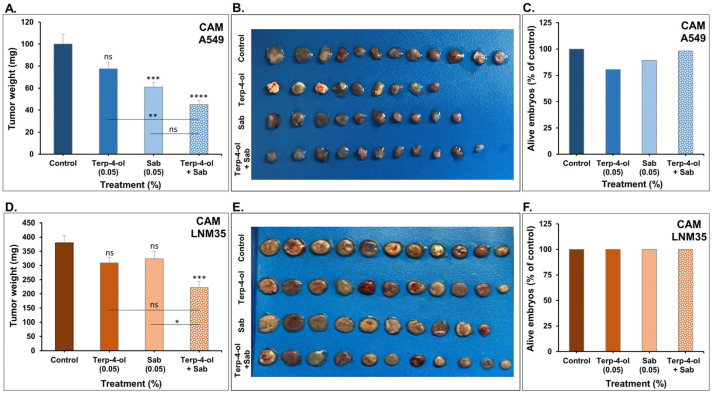
Effect of the combination of terpinen-4-ol and sabinene hydrate on NSCLC tumor growth in CAM. (**A**,**B**) Weight of A549 tumors xenografted on CAM and treated with or without terpinen-4-ol, sabinene hydrate, or a combination for 1 week. (**C**) Percentage of alive embryos in the A549 control and treated groups. (**D**,**E**) Weight of LNM35 tumors xenografted on CAM and treated with or without terpinen-4-ol, sabinene hydrate, or a combination for 1 week. (**F**) Percentage of alive embryos in the LNM35 control and treated groups. Columns are means; bars are S.E.M. * *p* < 0.05, ** *p* < 0.01, *** *p* < 0.001, and **** *p* < 0.0001. ns—non-significant.

To confirm the pharmacological relevance of this combined mixture, we assessed its impact on NSCLC tumors xenografted on chick embryo CAM. As shown in Figure 10, the combination significantly enhanced the effect of each of the monoterpenes by inhibiting A549 tumor growth by approximately 55%, compared to terpinen-4-ol or sabinene hydrate, which produced a 23% and 39% reduction in tumor weight, respectively (Figure 10A,B). This combination had a more evident effect on LNM35 tumors, inhibiting tumor growth by 42%, compared to non-significant reductions in tumor growth with the single treatments (Figure 10D,E). We determined toxicity by comparing the percentage of alive embryos in each group at day 17. The combination showed no cytotoxicity, as the percentage of alive embryos was similar between the control and the combined group (Figure 10C,F).

Previous research on these monoterpenes did not report their anticancer effect alone or in combination using the CAM model. Intravenous administration of terpinen-4-ol (0.06% and 0.1%) was reported to cause a dose-dependent reduction in tumor growth of A549 cells xenografted subcutaneously in BALB/c nu/nu mice [13]. Shapira et al. (2016) also reported that intra-tumoral injections of terpinen-4-ol (0.1% and 1%) significantly reduced tumor volume and weight in a colorectal mouse model without adverse effects [12].

## 4. Conclusions

Our findings highlight the promising anticancer potential of combining terpinen-4-ol with sabinene hydrate for the treatment of NSCLC. These findings pave the way for further pre-clinical studies investigating this combination across a wider spectrum of solid tumors and in diverse animal models, targeting various cancer hallmarks.

## Data Availability

Data are contained within the article.
